# Association between multiple vitamins and bone mineral density: a cross-sectional and population-based study in the NHANES from 2005 to 2006

**DOI:** 10.1186/s12891-023-06202-6

**Published:** 2023-02-10

**Authors:** Ruyi Zhang, Qin Huang, Guanhua Su, Muhong Wei, Yuan Cui, Haolong Zhou, Wenjing Song, Dongsheng Di, Junan Liu, Qi Wang

**Affiliations:** 1 MOE Key Lab of Environment and Health, Department of Epidemiology and Biostatistics, School of Public Health, Tongji Medical College, Huazhong University of Science and Technology, Wuhan, 430030 China; 2 Department of Rehabilitation Medicine, Union Hospital, Tongji Medical College, Huazhong University of Science and Technology, Wuhan, 430030 China; 3 Department of Cardiology, Union Hospital, Tongji Medical College, Huazhong University of Science and Technology, Wuhan, 430030 China; 4 Department of Social Medicine and Health Management, School of Public Health, Tongji Medical College, Huazhong University of Science and Technology, Wuhan, 430030 China

**Keywords:** Multiple vitamins co-exposure, Bone mineral density, Weighted quantile sum (WQS) regression, Principal components analysis (PCA)

## Abstract

**Background:**

Bone mineral density (BMD) alterations in response to multivitamin exposure were rarely studied. Our study assessed the association of coexposure to six types of vitamins (i.e., vitamins B12, B9, C, D, A and E) with BMD measurements in adults in the US.

**Methods:**

Data were collected from participants aged ≥ 20 years (*n* = 2757) in the U.S. National Health and Nutrition Examination Surveys (NHANES) from 2005 to 2006. Multiple linear regression, restricted cubic splines, principal component analysis (PCA) and weighted quantile sum (WQS) regression were performed for statistical analysis.

**Results:**

The circulating levels of vitamins B12 and C were positively associated with BMDs, and an inverted L-shaped exposure relationship was observed between serum vitamin C and BMDs. PCA identified two principal components: one for ‘water-soluble vitamins’, including vitamins B12, B9 and C, and one for ‘fat-soluble vitamins’, including vitamins A, D and E. The former was positively associated with total femur (*β* = 0.009, 95%CI: 0.004, 0.015) and femoral neck (*β* = 0.007, 95%CI: 0.002, 0.013) BMDs, and the latter was negatively associated with BMDs with non-statistical significance. The WQS index constructed for the six vitamins was significantly related to total femur (*β* = 0.010, 95%CI: 0.001, 0.018) and femoral neck (*β* = 0.008, 95%CI: 0.001, 0.015) BMDs, and vitamins B12 and C weighted the most. The WQS index was inversely related to BMDs with non-statistical significance, and vitamins E and A weighted the most.

**Conclusion:**

Our findings suggested a positive association between water-soluble vitamin coexposure and BMD, and the association was mainly driven by vitamins B12 and C. Negative association between fat-soluble vitamin coexposure and BMD was indicated, mainly driven by vitamins E and A. An inverted L-shaped exposure relationship was found between vitamin C and BMD.

**Supplementary Information:**

The online version contains supplementary material available at 10.1186/s12891-023-06202-6.

## Introduction

Osteoporosis is one of the most serious bone diseases affecting elderly individuals worldwide. The International Osteoporosis Foundation reported more than 8.9 million fractures due to osteoporosis every year globally [1]. Osteoporosis may cause unforeseen fractures and other distressful events, such as pain, disability and huge financial burden [2]. The mortality rate is up to 20%–40% in the first year after the occurrence of a hip fracture [3]. Many factors, including ageing, being female, disrupted estrogen metabolism and lack of physical activity, are associated with bone mineral density (BMD) reduction and contribute to osteoporosis pathogenesis [4, 5].

In recent years, the influence of diet nutrients and vitamins on bone health have been extensively investigated. A combination of vitamins D and K or supplementation of vitamin E alone inhibited bone loss in rats and mice [6, 7]. An in vitro study showed that high levels of vitamins C and E as antioxidants can promote the proliferation of bovine osteoblasts and support bone regeneration [8]. Several population-based studies revealed that elevated amount of vitamin C was associated with increased BMD and decreased risk of fracture [2, 9]. A study from the United States of America indicated that vitamins B12 and B9 were beneficial for osteoporosis prevention in the elderly women [10]. However, a recent randomized clinical trial performed on 2919 elderly individuals demonstrated no reduction in fracture risk after daily vitamin B supplementations [11]. One study in a mouse model showed that vitamin A promoted the proliferation of osteoclasts and thereby reduced BMD [12]. A cross sectional study including 6481 Korean adults found that vitamin A was associated with low BMD and increased fracture risk [13]. However, a 16-year randomized control trial observed no increase in fracture risk after long-term supplementation with high dose of vitamin A [14].

These results indicate that vitamins play critical roles in bone health maintenance. However, most studies have focused on a single or two vitamins [13, 15]. A drawback in these studies is the lack of understanding of the associations of multivitamin coexposure with bone health due to simultaneous exposure to multivitamins from diverse sources for individuals in reality. As far as we know, no study has explored the association between multiple vitamins and BMD, and our study is an attempt to fill the knowledge gap. Current studies have adopted traditional regression models to investigate vitamin and bone associations, and thus collinearity often occur, particularly regression coefficient sign reversal and variance inflation [10, 11]. Therefore, appropriate approaches, such as weighted quantile sum (WQS) regression modelling and principal component analysis (PCA), are needed in exploring the associations of multivitamin coexposure with BMD measurements and the role of each vitamin in vitamin–BMD associations. WQS regression can be used in assessing the dependency of an outcome on a weighted index indicating coexposure to multiple relevant vitamins. Using the idea of dimensionality reduction, the PCA can be used in developing a set of comprehensive indicators from multiple correlated vitamins and maximizing the coverage of original vitamin information. These methods have been gradually used in estimate the association of various multiple exposures, such as metals, and dietary carotenoid intake with bone health [16, 17].

In this study, we utilized the data of the US nationwide population from the National Health and Nutrition Examination Survey (NHANES) to study the individual and mixed associations of six vitamins (B12, B9, C, D, A and E) on BMD in US adults aged ≥ 20 years by using multiple linear regression, restricted cubic splines, PCA models and WQS regression.

## Materials and methods

### Study population

Data from the NHANES (https://www.cdc.gov/nchs/nhanes/index.htm) was used, which is a cross-sectional survey administered by the Health Statistics Center of American Centers for Disease Control and Prevention (CDC) to noninstitutionalized US residents. A stratified and multistage sampling design was adopted for the selection of representative samples. Baseline data on health and nutritional status were obtained by in-person interviews, mobile physical examination and laboratory testing. The NHANES was approved by the Ethics Review Committee of American National Health Statistics Center. Each participant provided informed consent.

**Fig. 1:**
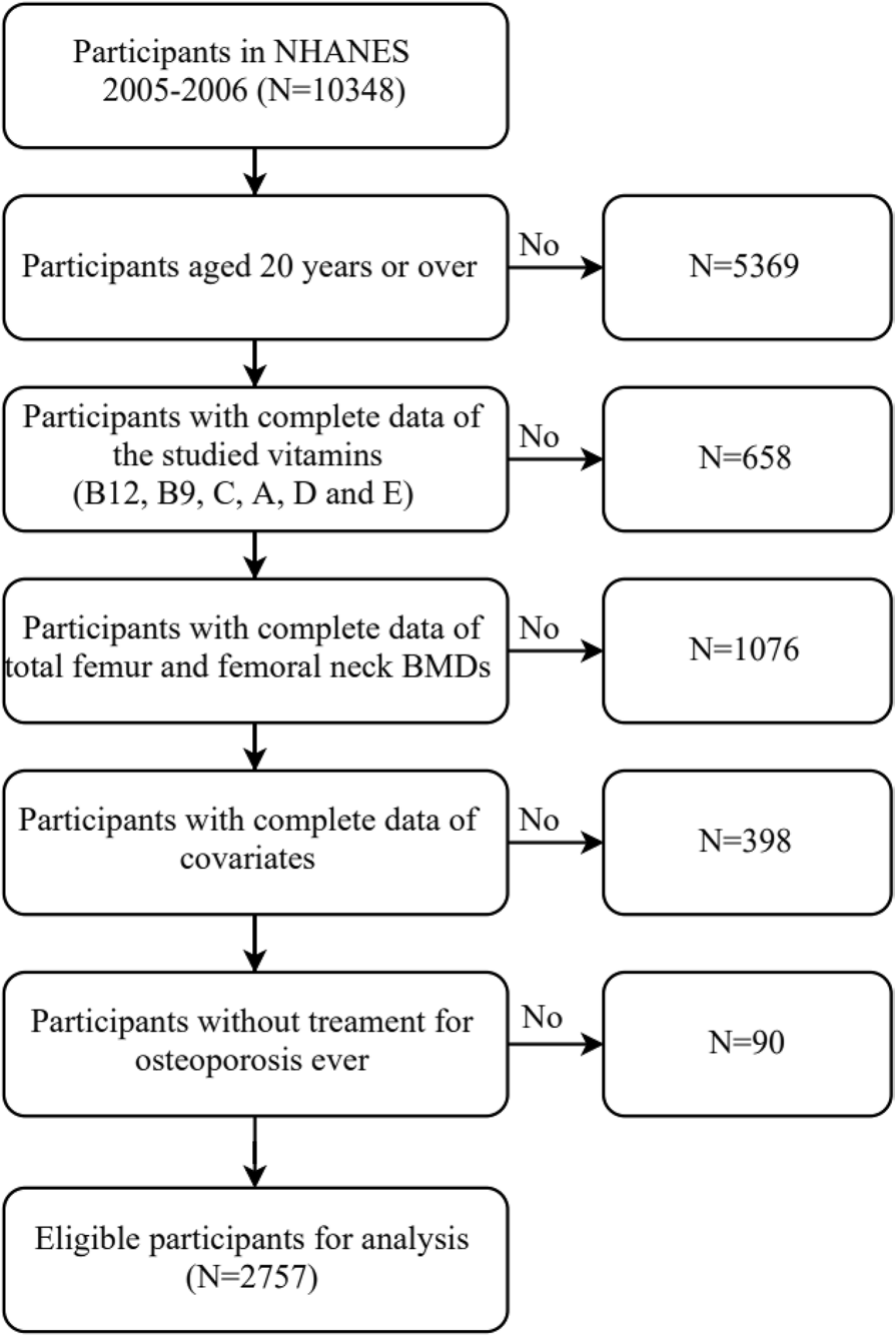
Flow chart for the selection of US population in the study

To date, the NHANES has been conducted for 11 rounds every 2 years since 1999. Given that the serum levels of the six vitamins, namely, B12, B9, C, D, A, and E, and the BMD measurement of the femur site were fully measured only in the NHANES 2005–2006, data from this cycle were used in this study. A total of 10,348 participants were included in the 2005–2006 cycle, and 5369 participants aged 20 years or below were excluded. In addition, we excluded 1734 participants who lacked information on BMD measurements and serum levels of the studied vitamins. Furthermore, we excluded 398 participants with missing covariates and 90 participants with osteoporosis treatments. Finally, a total of 2757 participants aged ≥ 20 years were included for the statistical analysis. The flow chart of participant exclusion is shown in Fig. 1.

### Vitamin measurements

Serum samples were collected in parallel with in-person interviews. And serum specimens were then processed, stored at –30 ℃ and shipped to the Division of Laboratory Sciences and CDC for analysis. The serum levels of the six vitamins were detected. Vitamin B12 and red blood cell folate (i.e., vitamin B9) were measured by using a ‘Quantaphase II Folate/Vitamin B12’ radioassay kit (Bio-Rad Laboratories) (https://wwwn.cdc.gov/Nchs/Nhanes/2005-2006/FOLATE_D.htm; https://wwwn.cdc.gov/Nchs/Nhanes/2005–2006/B12_D.htm). Vitamins A (retinol) and E (α-tocopherol) were measured by high-performance liquid chromatography (HPLC) with photodiode array detection (https://wwwn.cdc.gov/Nchs/Nhanes/2005-2006/VITAEC_D.htm). Vitamin C (ascorbic acid) was measured by isocratic HPLC with electrochemical detection at 650 mV1 (https://wwwn.cdc.gov/Nchs/Nhanes/2005-2006/VIC_D.htm). Serum 25-OH-D was measured by using an equilibrium radioimmunoassay procedure (https://wwwn.cdc.gov/Nchs/Nhanes/2005-2006/VID_D.htm). The process of vitamin measurements was described in detail in the NHANES laboratory manuals (https://wwwn.cdc.gov/nchs/nhanes/continuousnhanes/manuals.aspx?BeginYear=2005).

The limit of detection (LOD) for vitamins B12, B9, A, D and E were 20 pg/mL, 0.05 ng/mL, 1.03 μg/dL, 3.75 nmol/L, and 40.67 μg/dL, respectively. The lower limit of detection (LLOD) for vitamin C was 0.03 mg/dL in two rounds of NHANES. For the lower LOD or LLOD values, an imputed value of LOD/LLOD divided by the square root of 2 was adopted.

### BMD measurements

All participants received BMD measurements at the skeleton site of the femur via dual-energy X-ray absorptiometry (DXA) in the mobile examination center. Bone scans and scan analyses were performed using a Hologic QDR-4500A fan-beam densitometer (Hologic, Inc., Bedford, Massachusetts), and BMD measurement results were analyzed with Hologic Discovery v12.4 software (https://wwwn.cdc.gov/Nchs/Data/Nhanes/Dxa/DXXFEM_D.htm). Given that the total femur and femoral neck BMD measurements are relatively indicative of hip fracture and osteoporosis diagnosis in clinical setting, we used the total femur and femoral neck BMDs as outcome variables [18]. In the NHANES, BMD measurements were performed on the left femur and femoral neck region, unless answers to safety exclusion questions warrant the use of the right femoral neck.

### Covariates

Covariates in adjustment included age, sex, race, family poverty-to-income ratio (PIR), education, body mass index (BMI), calcium concentration, physical activity, drinking status, smoking status, regular milk consumption, osteoporosis family history, daily prednisone/cortisone intake, prevalent diseases of diabetes, and hypertension. They were selected as covariates according to the previous literature [19–23] and biological considerations. Sociodemographic factors (age, sex, race, family PIR, education, and osteoporosis family history), lifestyle factors (physical activity, drinking status, smoking status, regular milk consumption, and prednisone/cortisone intake daily), and disease status (prevalent diseases of diabetes, and hypertension) were obtained by in-person interviews. BMI data were obtained by physical examination, and calcium concentration was obtained by laboratory analysis.

Age was categorized into three groups (20–39, 40–59 and ≥ 60 years), and modelled as a continuous variable. Sex was categorized as male or female. Race was classified into five groups (Mexican American, Hispanic, non-Hispanic white, non-Hispanic black, and other races, including non-Hispanic multiracial). Education was classified into three groups (lower than high school, high school, and above high school). The family PIR was classified by poverty threshold (values 0 ~ 0.99 indicating below poverty and those ≥ 1.00 indicating as or above poverty) [19]. The BMI was calculated as weight (kg) divided by height squared (m^2^) and classified as no obese(< 30 kg/m^2^) and obese (≥ 30 kg/m^2^). Total calcium was measured by ion activity in solution with Beckman Synchron LX20 (https://wwwn.cdc.gov/Nchs/Nhanes/2005-2006/BIOPRO_D.htm), and was treated as a continuous variable. Physical activity was classified into four groups (sedentary = ‘no regular physical activity’; insufficient = ‘ < 500 metabolic equivalent (MET)-minutes per week’; moderate = ‘500–1000 metabolic equivalent (MET)-minutes per week’; and high = ‘ > 1000 metabolic equivalent (MET)-minutes per week’) according to the 2008 Physical Activity Guidelines for Americans [20]. Drinking status was categorized into three groups (current = ‘ > 12 alcohol drink in a year”; ever = ‘ < 12 alcohol drinks in a year but > 12 alcohol drinks in life’; and never = ‘ < 12 alcohol drinks in a year and in life’) [21]. Smoking status was categorized into three groups (current = ‘ > 100 cigarettes in life and smoked every day or some days during the survey time’; ever = ‘ > 100 cigarettes in life and did not smoke during the survey time at all’; and never = ‘ < 100 cigarettes in life’) [22] Regular milk consumption was assessed by the question ‘Have you use regular milk 5 times per week?’ (current, never, and ever). Family history of osteoporosis was assessed by the question ‘Have your parents ever told by a health professional that they had osteoporosis?’ (yes, no). Prednisone/cortisone intake daily was assessed by the question ‘Have you ever taken prednisone or cortisone daily?’ (yes, no) [23]. Prevalent diabetes (yes and no), and hypertension (yes and no) were assessed by the questions ‘Have doctors told you have diabetes?’ and ‘Have doctors ever told you had high blood pressure?’, respectively [23].

### Statistical analysis

The guidelines for the NHANES data analysis were retrieved from to (https://wwwn.cdc.gov/nchs/nhanes/tutorials/default.aspx). The summary statistics and distributions of BMD levels were computed for all the participants. Continuous variables were described by using mean and standard deviation (SD), and categorical variables were described by using frequencies and percentages. The concentrations of the six vitamins were described by using median and interquartile range (IQR) according to the variables of the participants’ characteristics. Differences in participants’ characteristics by sex were analyzed by using chi-square test (categorical variables) or t-test (continuous variables). Mutual correlations among the six vitamins were assessed by using Pearson correlation coefficients.

We used multiple linear regression to assess the dependency of total femur and femoral neck BMDs on each vitamin after adjusting all 15 covariates. Four groups by quartile (Q1, Q2, Q3, and Q4) were established for each vitamin, and the first group for each vitamin was used as the reference. Sampling weight was used in analyzing the data from complex survey. We also used restricted cubic splines to analyze the exposure relationship between log-transformed vitamins and BMDs.

#### WQS regression

WQS regression is an analytic method integrated with linear or logistic regression. It was used in estimating associations between multiple vitamin coexposure and BMD measurements [24]. The concentrations of the six vitamins were natural log-transformed because of their right-skewed distributions. WQS regression modelling consisted of two steps: i) the significance tests for the regression coefficient between the WQS index and outcome, and ii) the calculation of corresponding weights of important components in bootstrap samples. The weighted index was assessed with the following equation.

$$g\left(\mu \right)={\beta }_{0}+{\beta }_{1}\left(\sum_{i=1}^{c}{w}_{i}{q}_{i}\right)+{z}^{\mathrm{^{\prime}}}\varphi$$

Where ${\beta }_{0}$ and ${\beta }_{1}$ are the intercept and regression coefficient, respectively, ${z}^{^{\prime}}$ and $\varphi$ are the vector of covariates and its coefficient, respectively, and $c$ is the number of vitamins considered in the analysis (i.e., six in this study). The term $\sum_{i=1}^{c}{w}_{i}{q}_{i}$ represented the sum of the entire weighted index, and the weights were constrained to sum to 1. The variable $g\left(\mu \right)$ is a linear link function when outcomes were continuous (BMD). We split the data into two datasets (40% as training set and 60% as validation set), estimated the average of weights of each vitamin in the training dataset, and obtained ${\beta }_{1}$ in the validation dataset through 1000 times of bootstrapping.

We constrained the model was constrained to analyze the effect on outcomes in a single direction at one time. Given that we had no adequate knowledge of the directions of the multiple vitamins-BMD associations, we conducted the model with ${\beta }_{1}$ in positive and negative directions, and determined the two potentials of association directions. One of the directions was the assumption that the components of WQS index were all positively associated with BMD, whereas the other was the assumption that the components of WQS index were all negatively associated with BMD.

#### PCA

On the basis of a dimensionality reduction principle, PCA recombines several related original variables into a group of unrelated composite variables and extracts optimal composite variables to reflect original variable information as much as possible. PCA was used in deriving vitamin patterns on the basis of six log-transformed serum vitamins. A varimax rotation was applied to increase variance, obtain simple structure in the factor loading matrix and to save factor scores for each participant. To identify the number of components to be retained, we adopted the most widely used criterion (eigenvalues > 1.0). Two principal components (PCs) were retained for multiple linear regression analysis.

SAS 9.4 and R (4.0.4) were used for all statistical analyses. WQS regression was conducted with R packages ‘gWQS’ (version 3.0.3). All significance levels were set to less than 0.05.

## Results

### General characteristics of participants

The general characteristics of participants are presented in Table 1. A total of 2757 individuals (including 1230 women) were included in this study and had a mean age of 47.97 years. Men had a higher proportion of high physical activity and higher calcium level and were more likely to be alcohol drinkers, smokers and regular milk consumers compared with women (*P* value < 0.001). The mean BMD at the total femur and femoral neck was higher in men than in women (*P* value < 0.001). And Table S1 shows the overall distribution of six vitamins, and Table S2 shows the baseline levels of serum vitamins according to the baseline variables of the subjects.

**Table 1:** Characteristics of participants in the NHANES 2005–2006, stratified by sex

Characteristics	Total (*n* = 2757)	Men (*n* = 1527)	Women (*n* = 1230)	*P*
Age [yrs., mean ± SD]	47.97 ± 17.81	48.48 ± 18.18	47.34 ± 17.33	0.093
Age [yrs., n (%)]				
20 ~ 39	1000 (36.27)	557 (36.48)	443 (36.02)	0.012
40 ~ 59	949 (34.42)	493 (32.29)	456 (37.07)	
≥ 60	808 (29.31)	477 (31.24)	331 (26.91)	
Race [n (%)]				
Mexican American	557 (20.20)	318 (20.83)	239 (19.43)	0.126
Hispanic	88 (3.19)	47 (3.08)	41 (3.33)	
Non-Hispanic white	1416 (51.36)	792 (51.87)	624 (50.73)	
Non-Hispanic black	590 (21.40)	324 (21.22)	266 (21.63)	
Other	106 (3.84)	46 (3.01)	60 (4.88)	
Education [n (%)]				
Lower than high school	721 (26.15)	443 (29.01)	278 (22.60)	< 0.001
High school	646 (23.43)	364 (23.84)	282 (22.93)	
Above high school	1390 (50.42)	720 (47.15)	670 (54.47)	
PIR [mean ± SD]	2.80 ± 1.61	2.80 ± 1.61	2.81 ± 1.63	0.909
PIR [n (%)]				
0 ~ 0.99	440 (15.96)	245 (16.04)	195 (15.85)	0.892
≥ 1.00	2317 (84.04)	1282 (83.96)	1035 (84.15)	
BMI [kg/m^2^, mean ± SD]	27.76 ± 5.27	27.68 ± 4.63	27.86 ± 5.97	0.384
BMI [kg/m^2^, n (%)]				
≤ 30	1928 (69.93)	1105 (72.36)	823 (66.91)	0.002
≥ 30	829 (30.07)	422 (27.64)	407 (33.09)	
Physical activity [n (%)]				
Sedentary	994 (36.05)	554 (36.28)	440 (35.77)	< 0.001
Insufficient	584 (21.18)	287 (18.80)	297 (24.15)	
Moderate	348 (12.62)	187 (12.25)	161 (13.009)	
High	831 (30.14)	499 (32.68)	332 (26.99)	
Drink status [n (%)]				
Current	1998 (72.47)	1249 (81.79)	749 (60.89)	< 0.001
Ever	432 (15.67)	181 (11.85)	251 (20.41)	
Never	327 (11.86)	97 (6.35)	230 (18.70)	
Smoke status [n (%)]				
Current	666 (24.16)	416 (27.24)	250 (20.33)	< 0.001
Ever	708 (25.68)	471 (30.84)	237 (19.27)	
Never	1383 (50.16)	640 (41.91)	743 (60.41)	
Diabetes [n (%)]				
Yes	241 (8.74)	137 (8.97)	104 (8.46)	0.633
No	2516 (91.26)	1390 (91.03)	1126 (91.54)	
Hypertension [n (%)]				
Yes	825 (29.92)	455 (29.80)	370 (30.08)	0.871
No	1932 (70.08)	1072 (70.20)	860 (69.92)	
Regular milk consumption [n (%)]				
Current	1116 (40.48)	654 (42.83)	462 (37.56)	< 0.001
Never	601 (21.80)	294 (19.25)	307 (24.96)	
Ever	1040 (37.72)	579 (37.92)	461 (37.48)	
Prednisone/cortisone intake daily [n (%)]				
Yes	119 (4.32)	51 (3.34)	68 (5.53)	0.005
No	2638 (95.68)	1476 (96.66)	1162 (94.47)	
Osteoporosis family history [n (%)]				
Yes	248 (9.00)	99 (6.48)	149 (12.11)	< 0.001
No	2509 (91.00)	1428 (93.52)	1081 (87.89)	
Calcium [mmol/L, mean ± SD]	2.37 ± 0.09	2.38 ± 0.09	2.36 ± 0.09	< 0.001
Total femur BMD [gm/cm^2^, mean ± SD]	0.99 ± 0.16	1.04 ± 0.15	0.92 ± 0.15	< 0.001
Femoral neck BMD [gm/cm^2^, mean ± SD]	0.85 ± 0.15	0.88 ± 0.15	0.81 ± 0.14	< 0.001

*NHANES* National Health and Nutrition Examination Survey, *SD* standard deviation, *BMI* body mass index, *PIR* Family poverty-to-income ratio, *BMD*, bone mineral density

The mutual correlations among each two of the six vitamins were all statistically significant, except the correlation between vitamins A and B12, with a correlation coefficient (*r*) ranging from 0.03 to 0.41 (*P* value < 0.001, Fig. S1).

### Association between each vitamin and BMD measurements

We assessed the association between the circulating level of each vitamin and BMDs through multivariable linear regression analysis (Table 2). High vitamin B12 quartiles were positively associated with total femur (β _Q4 *VS* Q1_ = 0.021, 95% CI: 0.006, 0.036; *P* value = 0.005) and femoral neck (β _Q4 *VS* Q1_ = 0.021, 95% CI: 0.007, 0.035; *P* value = 0.003) BMDs after adjusting all the mentioned covariates. High vitamin C quartiles were positively associated with total femur (β _Q4 *VS* Q1_ = 0.024, 95% CI: 0.009, 0.040; *P* value = 0.002) and femoral neck (β _Q4 *VS* Q1_ = 0.020, 95% CI: 0.005, 0.034; *P* value = 0.009) BMDs. The associations of vitamins B9, A, D and E levels with BMD measurements did not achieve significance.

**Table 2:** Associations between the six vitamins and BMDs among participants in NHANES 2005–2006

Vitamins	Total femur BMD	*P*	Femoral neck BMD	*P*	Vitamins	Total femur BMD	*P*	Femoral neck BMD	*P*
B12 (pmol/L)					D (nmol/L)				
Q1	ref		ref		Q1	ref		ref	
Q2	0.010 (-0.004, 0.024)	0.167	0.010 (-0.003, 0.024)	0.139	Q2	-0.004 (-0.021, 0.012)	0.592	-0.009 (-0.024, 0.007)	0.273
Q3	**0.017 (0.002, 0.031)**	**0.022**	0.013 (-0.001, 0.027)	0.058	Q3	0.006 (-0.010, 0.022)	0.442	0.004 (-0.012, 0.020)	0.616
Q4	**0.021 (0.006, 0.036)**	**0.005**	**0.021 (0.007, 0.035)**	**0.003**	Q4	0.010 (-0.006, 0.026)	0.219	-0.001 (-0.017, 0.014)	0.818
B9 (nmol/L)					A (μmol/L)				
Q1	ref		ref		Q1	ref		ref	
Q2	-0.005 (-0.020, 0.010)	0.537	-0.007 (-0.022, 0.007)	0.348	Q2	0.001 (-0.015, 0.017)	0.886	-0.002 (-0.018, 0.013)	0.763
Q3	0.001 (-0.015, 0.017)	0.871	-0.003 (-0.018, 0.012)	0.724	Q3	-0.001 (-0.016, 0.015)	0.978	-0.004 (-0.019, 0.012)	0.642
Q4	0.007 (-0.008, 0.023)	0.370	0.001 (-0.014, 0.016)	0.876	Q4	0.007 (-0.009, 0.023)	0.391	-0.001 (-0.016, 0.014)	0.891
C (μmol/L)					E (μmol/L)				
Q1	ref		ref		Q1	ref		ref	
Q2	**0.024 (0.009, 0.040)**	**0.002**	**0.023 (0.008, 0.038)**	**0.002**	Q2	-0.004 (-0.019, 0.012)	0.652	-0.003 (-0.018, 0.012)	0.704
Q3	**0.027 (0.012, 0.042)**	** < 0.001**	**0.016 (0.002, 0.030)**	**0.023**	Q3	-0.001 (-0.018, 0.017)	0.939	-0.007 (-0.023, 0.009)	0.372
Q4	**0.024 (0.009, 0.040)**	**0.002**	**0.020 (0.005, 0.034)**	**0.009**	Q4	-0.001 (-0.017, 0.017)	0.978	-0.010 (-0.027, 0.007)	0.251

All estimated results were expressed as β (95%CI); β: partial regression coefficient; CI, confidence interval. Models were adjusted for age, sex, race, family poverty-to-income ratio, education, body mass index, calcium concentration, physical activity, drinking status, smoking status, regular milk consumption, osteoporosis family history, prednisone/cortisone intake daily, diabetes, and hypertension. BMD: bone mineral density. Boldness indicates a statistical significance

**Fig. 2:**
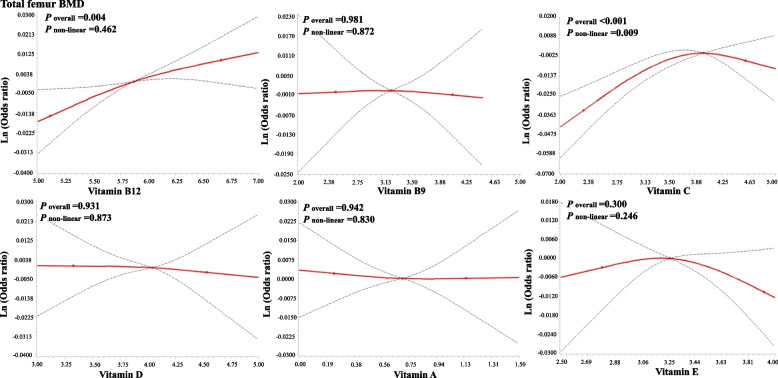
Adjusted exposure–response relationship between log-transformed vitamins and total femur BMD

**Fig. 3:**
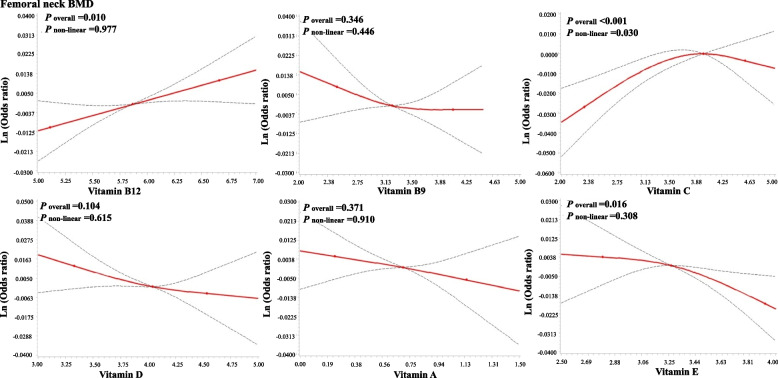
Adjusted exposure–response relationship between log-transformed vitamins and femoral neck BMD

Figures 2 and 3 present the exposure–response relationship between serum vitamin and BMDs. An inverted L-shaped exposure–response relationship was observed for vitamin C and total femur, and femoral neck BMDs (*P* for nonlinearity < 0.05).

### Multiple vitamin coexposure and BMD association assessed by PCA

Table S3 shows the factor loading matrix for the two retained PCs. We treated the two PCs as continuous variables and fitted the multiple linear regression to assess their associations with BMDs. The results are shown in Table 3. The factor 1, which had heavy loadings on ‘fat-soluble vitamins’ as vitamins E, A, and D, was associated with decreased total femur (*β* = 0.002, 95% CI: − 0.004, 0.008; *P* value = 0.480) and femoral neck (*β* =  − 0.004, 95% CI: − 0.010, 0.002; *P* value = 0.161) BMDs, although the effect values did not reach statistical significance. The factor 2, which had heavy loadings on the ‘water-soluble vitamins’ as vitamins B12, B9, and C, was associated with increased total femur (*β* = 0.009, 95% CI: 0.004, 0.015; *P* value = 0.001) and femoral neck (*β* = 0.007, 95% CI: 0.002, 0.013; *P* value = 0.005) BMDs.

**Table 3:** Association between the two major principal component and BMDs among participants in NHANES 2005–2006

Vitamin patterns	Outcome	β	95%CI	*P*
Factor 1: Fat-soluble vitamins	Total femur BMD	0.002	(-0.004, 0.008)	0.480
	Femoral neck BMD	-0.004	(-0.010, 0.002)	0.161
Factor 2: Water-soluble vitamins	Total femur BMD	0.009	(0.004, 0.015)	0.001
	Femoral neck BMD	0.007	(0.002, 0.013)	0.005

Models were adjusted for age, sex, race, family poverty-to-income ratio, education, body mass index, calcium concentration, physical activity, drinking status, smoking status, regular milk consumption, osteoporosis family history, prednisone/cortisone intake daily, diabetes, and hypertension

### Multiple vitamin coexposure and BMD association assessed by WQS regression

Table 4 summarizes the results of the WQS regression analysis of covariate-adjusted associations between multivitamin coexposure and BMD measurements. Specifically, the positive associations of statistical significance were found for the WQS indexes composed by the circulating levels of the six vitamins and total femur (*β*_*WQS*_ = 0.010, 95% CI: 0.001, 0.018; *P* value = 0.021) and femoral neck (*β*_*WQS*_ = 0.008, 95% CI: 0.001, 0.015; *P* value = 0.022) BMDs.

**Table 4:** Associations between the weighted quantile sum regression indexes composed for multiple vitamins and BMDs by WQS among participants in NHANES 2005–2006

	Outcome	β_WQS_	95%CI	*P*
Positive	Total femur BMD	0.010	(0.001, 0.018)	0.021
	Femoral neck BMD	0.008	(0.001, 0.015)	0.022
Negative	Total femur BMD	-0.003	(-0.011, 0.005)	0.457
	Femoral neck BMD	-0.007	(-0.015, 0.001)	0.099

Models were adjusted for age, sex, race, family poverty-to-income ratio, education, body mass index, calcium concentration, physical activity, drinking status, smoking status, regular milk consumption, osteoporosis family history, prednisone/cortisone intake daily, diabetes, and hypertension

**Fig. 4:**
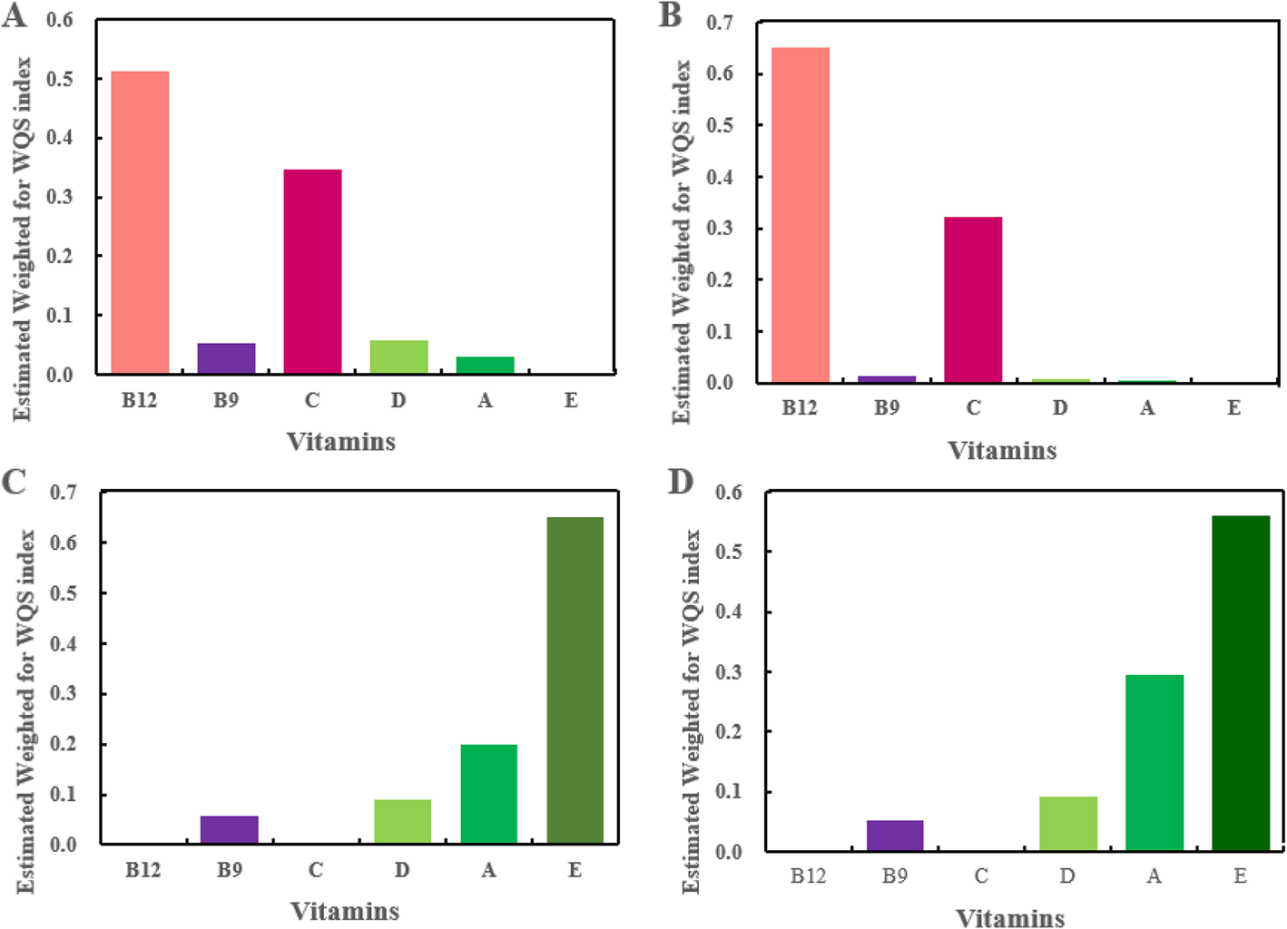
The WQS regression estimated weights of each of the six vitamins associated with total femur BMD (**A**) and femoral neck BMD (**B**) in the positive direction and total femur BMD (**C**) and femoral neck BMD (**D**) in the negative direction

The estimated weights for vitamins in composing BMD-associated WQS indexes are illustrated in Table S4 and Fig. 4. Vitamins B12 and C were predominant members for the positive association with total femur BMD (weights of 0.51 and 0.35, respectively) and femoral neck (weights of 0.65 and 0.32, respectively). Vitamins B12, C, and B9 were predominant members (weights of 0.47, 0.35, and 0.14, respectively) for the positive association with LS BMD, although of no significance was found. Vitamins E and A weighted the most in the multivitamin coexposure and total femur (weights of 0.65 and 0.20, respectively) and femoral neck (weights of 0.56 and 0.29, respectively) BMDs association, although of no significance was observed.

## Discussion

This study mainly aimed to determine the associations of coexposure with multiple vitamins (i.e., B12, B9, C, D, A, and E) and DXA-derived BMD measurements in US adults included in NHANES 2005–2006. Multiple linear regression analysis showed the positive associations of circulating vitamins B12 and C with BMDs, and an inverted L-shaped exposure–response relationship was observed between vitamin C and BMDs. WQS regression and PCA revealed a significant positive association between coexposure to water-soluble vitamins and BMDs, and the association was mainly driven by vitamins B12 and C. A negative association was found between coexposure to fat-soluble vitamins and BMDs, and was mainly driven by vitamins E and A.

### Water-soluble vitamins and BMD

Water-soluble vitamins consist of vitamin B and C family members. The positive associations between vitamin B members (B12 and B9) and BMD were consistent with the results of previous studies [10]. WQS analysis showed that vitamin B12 had the highest weight in the positive associations between multivitamins and BMDs, indicating that vitamin B12 may have a beneficial effect on bone health. A randomized trial reported that supplementation with a high-dose mixture of B vitamins contributed to elevated BMDs [25]. Vitamin B was a cofactor in homocysteine metabolism, and vitamin B12 and B9 deficiency caused elevated plasma homocysteine, which may interfere with collagen cross-linking, and damage the bone matrix and result in decreased BMD [26]. Previous trial in adults showed that treatment with B vitamins can decrease plasma homocysteine levels [27]. A previous study found that no association between vitamin B after adjusting for vitamins C and A, inconsistent with our findings [28]. Assumptions showed that they may indirectly regulate bone metabolism by acting on other vitamins. Several studies reported that vitamin B supplementation had no effect that lowered homocysteine level or changed bone markers [11, 29]. However, these studies have small sample size and restricted subjects.

Also known as ascorbic acid, vitamin C is a water-soluble vitamin. In the present study, we found that vitamin C contributes greatly and positively to the association of multivitamin coexposure with BMDs. To date, many studies have demonstrated that vitamin C contributes to elevated BMDs [9, 15]. Vitamin C can stimulate types 1 and 2 collagen synthesis, limit the lifetimes of osteoclasts, and prevent the loss of osteoblast markers [30]. Moreover, vitamins C and D can synergistically enhance osteocalcin (OC) promoter activity and carboxylated OC synthesis and thereby improve bone mass [28]. However, a recent study found a strong correlation between vitamin C and OC after adjusting serum vitamin D, indicating that vitamin C plays a more direct and important role in OC [31]. Vitamin C, which has strong biological activity, promotes the absorption of calcium in the body, and alleviates the toxicity of external toxic substances, is a nutrient that benefits bone metabolism [2]. Clinically, vitamin C deficiency causes scurvy and a range of skeletal symptoms, such as osteoporosis [32]. We found an inverted L-shaped exposure–response relationship between vitamin C and BMD. This association may be related to oxalate accumulation due to a high level of vitamin C. A case report showed that a high level of vitamin C induced oxalate accumulation, and resulted in bone pain [33]. Differences among these studies may be related to the dual roles of vitamin C in osteoclasts, and thus more studies must be performed.

### Fat-soluble vitamins and BMD

All the three statistical methods suggested significant negative association between vitamin E and BMD, consistent with the previous study [34]. WQS analysis shows that vitamin E had the highest weight in the negative associations between multivitamins and BMDs, indicating that vitamin E may have a damaging effect on bone health. In our study, a-tocopherol levels in sera were detected as the biomarkers of internal exposure to vitamin E. In mammals, a-tocopherol is the dominant isoform of vitamin E and is the major form of vitamin E supplements in the U.S. [35]. Serum a-tocopherol improved osteoclast activity, induced osteoclast fusion and differentiation and exerted an unfavorable effect on the bone [6]. However, a study with inconsistent conclusions showed that a-tocopherol was positively associated with BMD [36]. Therefore, studies are warranted to determine the association of vitamin E and its isomers with bone health.

As the main fat-soluble vitamins, vitamins A and D play interactive functions with each other. The binding of vitamin D receptor (VDR) and nuclear retinoid X receptor (RXR) forms the VDR/RXR complex, which further combines with vitamin D to exert vitamin D’s effect [28]. The RXR is also the receptor of vitamin A, and elevated levels of vitamin A may compete with vitamin D to bind to the VDR/RXR complex, thereby inhibiting vitamin D’s effect [13, 37]. On the one hand, WQS analysis and PCA revealed suggestive negative association of vitamin A with BMD, which is consistent with some epidemiologic studies [37, 38]. The possible mechanisms may be that its metabolic intermediate retinoic acid can increase osteoclast proliferation and enhance bone resorption, which weakens the bone [12]. On the other hand, our study found no significant positive association of serum vitamin D with BMD. To the best of our knowledge, vitamin D plays an important role in BMD by regulating calcium and phosphorus status and promoting bone mineralization [39]. Vitamin D deficiency can lead to osteomalacia [40], which would lead to decreased BMD. Meanwhile, a 1-year randomized control trial have reported that a dietary pattern with vitamin D reduced bone loss in the elderly with osteoporosis [41]. The difference in our results may be due to the inhibitory effect of vitamin A on vitamin D. Given that they can bind to osteocalcin-related receptor RXR [28], a competitive receptor relationship antagonizes some actions with each other. One study provided evidence that excessive VA may inhibit the favorable effect of vitamin D on BMD and potentiate the vitamin D deficiency–insufficiency effect [42]. Vitamins A and D are mutually restricted. Patients with severe vitamin D deficiency should avoid excessive intake of vitamin A when taking vitamin D as supplementary therapy. Interestingly, a 4-year cohort study found an inverted U-shaped association between vitamin A and BMD, indicating that low level of vitamin A reduces BMD [43]. Therefore, vitamin A may play dual roles on bone metabolism and exerts a positive effect on BMD at low levels and an inhibition effect on vitamin D at high levels. Thus, more studies are needed to determine its optimal level.

### Strengths and limitations

Our study has several strengths. The WQS regression and PCA methods were used in determining the associations of multivitamin coexposure and BMD measurements, accounting for the collinearity of multivitamins. The WQS approach was used in evaluating potentially important contributors to the association between multivitamin co-exposure and BMD. Serum vitamin concentrations were used to represent vitamin exposure levels, which was more accurate and indicative of internal exposure level than food-frequency questionnaire survey.

Our study has some potential limitations. We used the data from a cross sectional survey, and thus result on the causal inference of multivitamin exposure on BMD was limited. Large prospective cohort studies and experimental studies are needed to accumulate evidence. We included six types of vitamins in this study and omitted other vitamins (e.g., vitamin K) due to the absence of eligible data. US participants were studied, and the generalization of our findings to other ethnic populations is limited.

## Conclusion

The associations between exposure to multiple vitamins and BMDs were explored by using generalized linear regression, restricted cubic splines, WQS regression, and PCA methods with the NHANES 2005–2006 data of US adults aged ≥ 20 years. Vitamin B12 and C levels were associated positively, and an inverted L-shaped exposure–response relationship was observed between vitamin C and BMD. The water-soluble vitamins were significantly associated with increased BMDs, and association was mainly driven by B12 and C. The fat-soluble vitamins were associated with decreased BMDs, and the association was mainly driven by vitamin E and A. Our study provided evidence of the associations between multiple vitamin coexposure and BMDs.

## Supplementary Information

**Additional file 1:** **Fig.S1.** Pearson correlations among blood concentrations of the six studied vitamins (B12, B9,C, D, A and E) in this research. All *P* values <0.001 except for thecorrelation between vitamins A and B12. **Table S1.** Distribution of concentrationof the six studied vitamins among participants in NHANES 2005-2006. **Table S2.** Distribution of concentrationof the six studied vitamins accordingto the characteristics of participants in the NHANES 2005–2006.**Table S3.** Orthogonal rotated factor-loading matrix for serumvitamins among participants in NHANESs 2003-2006.**Table S4.** Weight of vitamins in WQS regression for the associationswith BMDs among participants in NHANES 2005-2006.

## Data Availability

Publicly available datasets were analyzed in this study. This data can be found here: https://www.cdc.gov/nchs/nhanes/index.htm
