# Supplementary material for: Association between multiple vitamins and bone mineral density: a cross-sectional and population-based study in the NHANES from 2005 to 2006
Source: BMC Musculoskelet Disord. 2023 Feb 10;24:113. doi: 10.1186/s12891-023-06202-6 (PMC9912521; doi:10.1186/s12891-023-06202-6)
Supplement: Supplementary file 1 — Additional file 1: Fig.S1. Pearson correlations among blood concentrations of the six studied vitamins (B12, B9,C, D, A and E) in this research. All P values <0.001 except for thecorrelation between vitamins A and B12. Table S1. Distribution of concentrationof the six studied vitamins among participants in NHANES 2005-2006. Table S2. Distribution of concentrationof the six studied vitamins accordingto the characteristics of participants in the NHANES 2005–2006.Table S3. Orthogonal rotated factor-loading matrix for serumvitamins among participants in NHANESs 2003-2006.Table S4. Weight of vitamins in WQS regression for the associationswith BMDs among participants in NHANES 2005-2006. [file 12891_2023_6202_MOESM1_ESM.docx]

**Supplementary materials**

**Association between multiple vitamins and bone mineral density: A population-based study in the NHANESs from 2005 to 2006**

Ruyi Zhang^1,#^, Qin Huang^3,#^, Guanhua Su^4,#^, Muhong Wei^1^, Yuan Cui^1^, Haolong Zhou^1^, Wenjing Song^1^, Dongsheng Di^1^, Junan Liu^2,*^, Qi Wang^1,*^

1 MOE Key Lab of Environment and Health, Department of Epidemiology and Biostatistics, School of Public Health, Tongji Medical College, Huazhong University of Science and Technology, Wuhan 430030, China

2 Department of Social Medicine and Health Management, School of Public Health, Tongji Medical College, Huazhong University of Science and Technology, Wuhan 430030, China

3 Department of Rehabilitation Medicine, Union Hospital, Tongji Medical College, Huazhong University of Science and Technology, Wuhan 430030, China

4 Department of Cardiology, Union Hospital, Tongji Medical College, Huazhong University of Science and Technology, Wuhan 430022, China

#These authors contributed equally to this article.

*Correspondence: +86-02783692701 [wangqi_tj@hust.edu.cn](mailto:wangqi_tj@hust.edu.cn)

The name of journal: **BMC Musculoskeletal Disorders**

**Supplementary legend**

**Fig. S1** Pearson correlations among blood concentrations of the six studied vitamins (B12, B9, C, D, A and E) in this research. All *P* values <0.001 except for the correlation between vitamins A and B12.

**Table S1** Distribution of concentration of the six studied vitamins among participants in NHANES 2005-2006.

**Table S2** Distribution of concentration of the six studied vitamins according to the characteristics of participants in the NHANES 2005–2006.

**Table S3** Orthogonal rotated factor-loading matrix for serum vitamins among participants in NHANESs 2003-2006.

**Table S4** Weight of vitamins in WQS regression for the associations with BMDs among participants in NHANES 2005-2006.


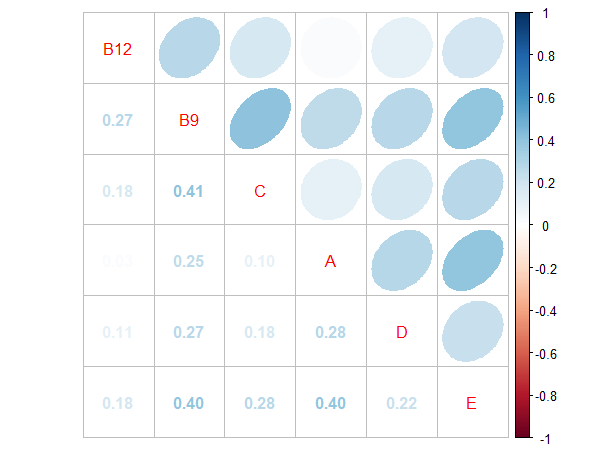


**Fig. S1** Pearson correlations among blood concentrations of the six studied vitamins (B12, B9, C, D, A and E) in this research. All *P* values <0.001 except for the correlation between vitamins A and B12.

**Table S1** Distribution of concentration of the six studied vitamins among participants in NHANES 2005-2006.

| Vitamins | GM±GSD | Percentiles | | | | |
| --- | --- | --- | --- | --- | --- | --- |
|  |  | 5th | 25th | 50th | 75th | 95th |
| B12 (pmol/L) | 356.73±3.52 | 165.31 | 261.99 | 352.76 | 476.01 | 782.28 |
| B9 (nmol/L) | 26.21±0.25 | 12.20 | 18.80 | 26.00 | 35.60 | 59.60 |
| C (μmol/L) | 45.45±0.59 | 10.20 | 36.30 | 54.50 | 69.30 | 97.70 |
| D (nmol/L) | 53.81±0.38 | 27.80 | 42.20 | 56.80 | 71.30 | 93.20 |
| A (μmol/L) | 2.01±0.01 | 1.27 | 1.69 | 2.03 | 2.41 | 3.12 |
| E (μmol/L) | 27.03±0.18 | 16.254 | 21.11 | 26.24 | 32.97 | 50.62 |

GM, geometric mean; GSD, geometric standard deviation.

**Table S2** Distribution of concentration of the six studied vitamins according to the characteristics of participants in the NHANES 2005–2006.

| Characteristics | Vitamin B12  (pmol/L) | Vitamin B9  (nmol/L) | Vitamin C  (μmol/L) | Vitamin A  (μmol/L) | Vitamin D  (nmol/L) | Vitamin E  (μmol/L) |
| --- | --- | --- | --- | --- | --- | --- |
| Sex |  |  |  |  |  |  |
| Male | 349.81 (195.57) | 24.70 (15.60) | 50.50 (33.50) | 2.13 (0.69) | 56.80 (26.70) | 26.24 (11.70) |
| Female | 358.30 (242.80) | 27.40 (18.20) | 58.50 (34.70) | 1.89 (0.75) | 56.80 (29.10) | 26.24 (12.47) |
| Age [yrs.] |  |  |  |  |  |  |
| 20~39 | 347.60 (196.67) | 23.60 (13.25) | 52.80 (28.40) | 1.91 (0.69) | 56.80 (29.10) | 22.67 (8.17) |
| 40~59 | 352.76 (202.21) | 25.40 (15.40) | 52.80 (34.60) | 2.01 (0.70) | 54.40 (26.70) | 27.40 (10.68) |
| ≥60 | 361.62 (247.60) | 31.70 (21.50) | 59.90 (38.00) | 2.22 (0.80) | 56.80 (26.60) | 30.88 (15.56) |
| Race |  |  |  |  |  |  |
| Mexican American | 352.76 (210.33) | 23.10 (12.00) | 51.70 (29.50) | 1.93 (0.66) | 51.90 (19.40) | 25.77 (11.36) |
| Hispanic | 345.76 (189.31) | 26.05 (12.25) | 56.20 (28.70) | 1.86 (0.70) | 49.50 (21.90) | 25.31 (8.17) |
| Non-Hispanic white | 338.00 (203.68) | 28.80 (19.70) | 56.20 (39.10) | 2.17 (0.75) | 64.10 (25.50) | 27.86 (13.31) |
| Non-Hispanic black | 378.59 (239.85) | 23.30 (13.90) | 53.90 (27.90) | 1.86 (0.73) | 39.80 (21.70) | 22.97 (8.99) |
| Other | 369.00 (199.26) | 25.70 (15.80) | 57.05 (34.10) | 1.91 (0.81) | 54.40 (26.70) | 26.12 (13.05) |
| Education |  |  |  |  |  |  |
| Lower than high school | 362.36 (227.31) | 23.60 (14.10) | 49.40 (32.90) | 1.99 (0.75) | 51.90 (24.30) | 25.54 (11.63) |
| High school | 343.91 (204.43) | 25.80 (17.70) | 51.10 (35.20) | 1.99 (0.73) | 54.40 (26.70) | 25.08 (11.82) |
| Above high school | 352.76 (211.06) | 27.40 (17.20) | 57.90 (31.30) | 2.06 (0.72) | 59.20 (29.10) | 26.94 (12.12) |
| Family poverty-to-income ratio |  |  |  |  |  |  |
| 0~0.99 | 360.51 (201.85) | 23.70 (14.20) | 49.10 (33.80) | 1.88 (0.71) | 51.90 (25.50) | 23.92 (9.78) |
| ≥ 1.00 | 351.29 (214.76) | 26.50 (17.50) | 55.60 (32.90) | 2.06 (0.73) | 56.80 (29.10) | 26.70 (12.17) |
| BMI [kg/m^2^] |  |  |  |  |  |  |
| < 30 | 359.41 (218.08) | 26.50 (17.20) | 56.80 (33.50) | 2.04 (0.73) | 59.20 (26.60) | 25.77 (12.13) |
| ≥30 | 335.05 (200.00) | 24.70 (15.20) | 50.00 (31.30) | 2.01 (0.73) | 51.90 (26.60) | 26.94 (11.19) |
| Physical activity |  |  |  |  |  |  |
| Sedentary | 353.50 (210.33) | 24.50 (15.40) | 48.80 (36.40) | 2.00 (0.74) | 51.90 (26.70) | 25.77 (11.91) |
| Insufficient | 349.81 (210.33) | 25.90 (17.30) | 55.60 (31.20) | 2.00 (0.75) | 54.40 (26.70) | 26.01 (11.15) |
| Moderate | 347.97 (197.05) | 28.50 (18.05) | 57.60 (32.95) | 2.09 (0.72) | 59.20 (26.70) | 26.82 (11.95) |
| High | 354.24 (221.40) | 26.70 (16.50) | 58.50 (30.00) | 2.06 (0.72) | 59.20 (29.10) | 26.47 (12.31) |
| Drink status |  |  |  |  |  |  |
| Current | 345.75 (200.74) | 25.80 (17.00) | 53.90 (32.90) | 2.08 (0.72) | 56.80 (26.60) | 26.47 (11.87) |
| Ever | 374.17 (241.70) | 26.15 (17.60) | 55.10 (33.20) | 1.92 (0.74) | 51.90 (24.30) | 25.66 (12.31) |
| Never | 360.88 (242.06) | 27.20 (16.80) | 56.80 (33.50) | 1.85 (0.85) | 51.90 (26.70) | 24.85 (11.87) |
| Smoke status |  |  |  |  |  |  |
| Current | 326.93 (181.55) | 22.40 (13.50) | 42.60 (42.10) | 2.00 (0.69) | 54.40 (31.50) | 23.92 (9.82) |
| Ever | 352.76 (219.92) | 27.60 (18.70) | 57.30 (33.50) | 2.17 (0.81) | 59.20 (26.60) | 28.79 (13.50) |
| Never | 366.05 (222.87) | 26.70 (16.90) | 57.30 (29.00) | 1.98 (0.72) | 54.40 (26.70) | 26.01 (12.24) |
| Diabetes |  |  |  |  |  |  |
| Yes | 366.79 (258.30) | 27.90 (16.10) | 48.30 (36.90) | 2.20 (0.83) | 49.50 (24.10) | 27.40 (15.14) |
| No | 352.40 (206.64) | 25.80 (17.00) | 55.10 (32.40) | 2.02 (0.72) | 56.80 (29.10) | 26.01 (11.68) |
| Hypertension |  |  |  |  |  |  |
| Yes | 355.72 (225.09) | 28.10 (19.50) | 53.90 (31.80) | 2.22 (0.81) | 54.40 (26.70) | 28.79 (14.23) |
| No | 352.03 (204.79) | 25.10 (15.40) | 55.10 (33.25) | 1.96 (0.69) | 56.80 (29.10) | 25.31 (10.98) |
| Regular milk consumption |  |  |  |  |  |  |
| Current | 364.94 (214.39) | 27.40 (17.40) | 55.60 (31.20) | 2.09 (0.78) | 59.20 (26.70) | 26.47 (11.81) |
| Never | 329.89 (197.04) | 23.60 (14.50) | 51.70 (34.60) | 1.93 (0.73) | 51.90 (29.00) | 25.08 (10.26) |
| Ever | 354.24 (216.97) | 26.00 (16.90) | 55.60 (32.90) | 2.01 (0.71) | 54.40 (26.70) | 26.47 (12.70) |
| Prednisone/cortisone intake daily |  |  |  |  |  |  |
| Yes | 362.36 (268.63) | 26.30 (20.60) | 53.90 (39.20) | 2.14 (0.83) | 56.80 (29.10) | 27.40 (14.86) |
| No | 352.76 (211.80) | 26.00 (16.80) | 54.50 (32.40) | 2.02 (0.73) | 56.80 (26.70) | 26.24 (11.89) |
| Osteoporosis family history |  |  |  |  |  |  |
| Yes | 337.27 (178.59) | 27.40 (17.15) | 56.80 (34.90) | 2.02 (0.65) | 58.00 (27.90) | 27.86 (12.25) |
| No | 354.24 (217.71) | 25.80 (17.00) | 54.50 (32.90) | 2.03 (0.74) | 56.80 (26.70) | 26.01 (11.94) |
| Calcium [mmol/L] ^a^ |  |  |  |  |  |  |
| < 2.38 | 338.00 (207.38) | 25.50 (16.50) | 54.50 (32.90) | 1.97 (0.71) | 54.40 (26.70) | 25.77 (11.09) |
| ≥2.38 | 368.26 (216.97) | 26.70 (17.40) | 54.50 (31.80) | 2.11 (0.73) | 56.80 (26.60) | 26.94 (12.77) |
| Total BMD [gm/cm^2^] ^a^ |  |  |  |  |  |  |
| < 0.99 | 351.29 (226.57) | 27.20 (18.40) | 56.80 (36.30) | 2.02 (0.76) | 56.80 (26.60) | 26.94 (13.57) |
| ≥0.99 | 353.50 (197.05) | 24.90 (15.00) | 52.80 (30.60) | 2.03 (0.71) | 54.40 (26.70) | 25.54 (10.50) |
| LS BMD [gm/cm^2^] ^a^ |  |  |  |  |  |  |
| < 0.84 | 349.81 (221.40) | 27.60 (19.30) | 56.80 (37.40) | 2.07 (0.75) | 56.80 (26.60) | 28.10 (14.00) |
| ≥0.84 | 354.98 (202.21) | 24.50 (14.00) | 52.80 (29.50) | 1.98 (0.71) | 54.40 (29.10) | 24.61 (10.03) |

All estimated results were expressed as median (IQR); *IQR*, interquartile range; *NHANES*, National Health and Nutrition Examination Survey; *BMI*, body mass index; *BMD*, bone mineral density. The ^a^ indicate that variables were divided into two groups based on the median

**Table S3** Orthogonal rotated factor-loading matrix for serum vitamins among participants in NHANESs 2003-2006.

| Vitamin | Factor 1 | Factor 2 |
| --- | --- | --- |
| B12 | -0.097 | 0.735 |
| B9 | 0.426 | 0.657 |
| C | 0.192 | 0.683 |
| D | 0.610 | 0.136 |
| A | 0.837 | -0.087 |
| E | 0.651 | 0.350 |
| Eigenvalues | 2.238 | 1.074 |

**Table S4** Weight of vitamins in WQS regression for the associations with BMDs among participants in NHANES 2005-2006.

|  | Total femur BMD | Weights | Femoral neck BMD | Weights |
| --- | --- | --- | --- | --- |
| Positive | **B12** | **0.512181** | **B12** | **0.649690** |
|  | **C** | **0.347343** | **C** | **0.321686** |
|  | D | 0.058589 | B9 | 0.014781 |
|  | B9 | 0.051859 | D | 0.007305 |
|  | A | 0.029620 | A | 0.006170 |
|  | E | 0.000408 | E | 0.000368 |
|  |  |  |  |  |
| Negative | **E** | **0.650752** | **E** | **0.560594** |
|  | **A** | **0.198818** | **A** | **0.294373** |
|  | D | 0.088464 | D | 0.090829 |
|  | B9 | 0.058476 | B9 | 0.052909 |
|  | B12 | 0.002220 | C | 0.001194 |
|  | C | 0.001269 | B12 | 0.000101 |

WQS, weighted quantile sum; BMD: bone mineral density; Component weights reported as percentage for vitamins with significant effects. Boldness indicates a weight of >0.1.
